# Modelling and Investigation of Energy Harvesting System Utilizing Magnetically Levitated Permanent Magnet

**DOI:** 10.3390/s22176384

**Published:** 2022-08-24

**Authors:** Joanna Bijak, Tomasz Trawiński, Marcin Szczygieł, Zygmunt Kowalik

**Affiliations:** Department of Mechatronics, Faculty of Electrical Engineering, Silesian University of Technology, 44-100 Gliwice, Poland

**Keywords:** energy harvesting, magnetic spring, mathematical modelling, Denavit-Hartenberg notation

## Abstract

The aim of this article is mathematical modelling and investigation of chosen parameters of a small energy harvesting system designed for energy harvesting from car tire mechanical vibrations to provide power supply for various sensors, e.g., in tire pressure monitoring system. The energy harvester consists of three permanent magnets inserted into a tube made from polyamide material. Comsol program has been used to calculate the force between the magnets, the stiffness of the magnetic spring, and the natural frequency of the system. MATLAB program has been used to simulate the movement of the moveable magnet to compare it with the measurements. Finally, the parameters of the mathematical model of the energy harvester were investigated and validated on a specially prepared laboratory test bench.

## 1. Introduction

In recent years, the energy harvesters for portable devices, for example, smartwatches [1], for moveable systems like vehicles to provide power for sensors in tire pressure monitoring systems, from tires [2] and shock absorbers [3,4], for sport- or medicine- related sensors from the motion of human body [5] and for a hard-to-reach system like vibration sensors on bridges [6] are developed. One of such energy harvesters are electromagnetic generators. In the electromagnetic generator, according to Faraday’s law, whenever there is relative motion between the magnet and the coil (wound around the permanent magnets system), an electromotive force is induced in the coil. The electromotive force depends on the relative velocity between the magnet and coil, the magnetic flux density, the coil radius, and the coil area, so to maximize the energy harvested in that system it is possible to change these parameters. For example, to increase the flux density it is possible to replace a single magnet with two or more of them with some material with different permeability [7,8] between them. One of the disadvantages of such systems is that they should work in the resonant frequency in order to recover a significant amount of energy [9]. Such energy harvesters are the inertial generator, which means the proof-mass, which is a magnet, should be placed in the frame, in which it could freely move [9]. One of the possible solutions for the movement enabling is a magnetic spring. The magnetic spring is the main element of this electromagnetic energy harvester system we are going to focus on.

The magnetic spring consists of permanent magnets, where a moveable magnet is positioned and levitates, in this case, between two fixed magnets. Repulsive forces are acting between the moveable magnet and fixed magnets. These forces are used to create two passive spring forces acting on the movable magnet from opposite sides (from the top and from the bottom [7,10,11]. A magnetic spring is more durable than the mechanical one and can be designed towards lowering the natural resonance of the whole device [8,10]. The resonance of this system depends on the stiffness coefficient and the mass of the movable magnet, so on the magnets parameters, such as diameters, heights, and the distance between them. Magnetic springs are used in electromagnetic generators not only for the placement of the moveable magnet but also to adjust the resonance frequency [12,13,14]. The higher the mass of the movable magnet and the lower the stiffness coefficient, the lower the resonance frequency [7,8,15,16]. It is possible to obtain another resonance frequency by using other configurations of the magnetic spring, for example only two magnets [7], an unfixed top magnet [11], without the top magnet [8], or by using block magnets instead of cylindrical ones [15]. Decreasing the distance between the fixed magnets and the movable magnet results, generally, in increasing the resonant frequency of the energy harvester [12].

Models of the magnet movement in the magnetic spring are derived from Newton’s second law, where the movement of the magnet depends on the forces acting on it [12,17,18,19,20,21,22,23,24]. In the modeling of the magnet movement, authors are often focused on magnetic spring equation, not on the movement equation. In [12,17,18,19,20] Coulomb’s theorem was used to derive magnetic force equation. In [12,17] authors calculate two stiffness coefficients: linear and nonlinear analytically and the force is calculated from it. In [18] the magnetic force is directly used in the movement equation and the stiffness coefficients are not calculated. In [19] the stiffness coefficient was assumed to be variable and depending on the magnetic field intensity. In [20] curve fitting from FEA calculation was used additionally to calculate force. In [21] the force was calculated by subtraction of the repulsive force between two magnets and the force was derived from FEM calculation and 5th polynomial curve fitting. In [22] magnetic force was calculated using Maxwell’s tensor method. In [23] magnet spring was used as a negative stiffness spring in addition to mechanical spring, magnetic spring force was derived using Ampere law. In [24] magnetic spring force was derived from simulation in ANSYS Maxwell and fitted to an exponential curve. However, in each of mentioned papers, the general equation of the movement was the same and a linear movement of the magnet was assumed. Our goal was to show the possibility of the new mathematical model of the magnet movement application. The energy harvester is compared to one degree of freedom manipulator with a prismatic joint with springs and dampers. Such a comparison (to manipulator) allows us to determine the movement of a moveable magnet in a system with more degrees of freedom. For example, in such an energy harvester without an internal rod, the moveable magnet is rotating due to misalignment of the magnets in the real magnetic spring and in consequence of the torque from repulsive forces. The main movement is linear, but it is disturbed by rotational movement and additional friction force, when one of the edges of the moveable magnet is touching a tube. This model can be used in the future to determine the best parameters of permanent magnets when the desired movement of the moveable magnet and frequency is known.

In this paper, a mathematical model of a magnetic spring is considered for the simulation of an electromagnetic energy harvesting system and the results of computer simulations are compared with results obtained from tests research carried out in a laboratory. In Section 2, the model of electromagnetic energy harvester and the spring force for several cases of magnetic spring is introduced. The spring force was obtained using a FEM program. In Section 3, several cases of the magnetic spring parameters were investigated to show the possibility of resonance frequency adjustment through stiffness coefficient changes. Additionally, in Section 3 the laboratory stand and the magnetic spring prototype along with measurement results are described. The mathematical model is also shown in Section 3. In Section 4, the mathematical model of the magnetic spring on the vibration generator is described and the equation of the object’s movement is derived. The developed mathematical model can be used to determine the movement of the moveable magnet in the magnetic spring with given parameters. That model is derived from the Euler-Lagrange equation and the homogenous transformation used in the kinematics and dynamics of robots. It can lead to obtaining the proper value of the resonant frequency of the whole energy harvester, which allows us to reach possibly higher harvested energy. In Section 5, the Simulink model and parameters are described. The Simulink model is used to conduct a simulation of the movements. In Section 6, results from simulation and measurements are compared. Finally, Section 7 contains conclusions.

## 2. Magnetic Spring and Prototype of Energy Harvester

The electromagnetic energy harvester is a converter of mechanical kinetic energy into electric energy, which uses Lorentz law to achieve this. In the case of rotary and swing [25] motions, the electromagnetic energy harvester may use an electric energy generator consisting of single or multiple movable coils placed between permanent magnets, similar to the construction of a voice coil motor used in hard disk drives for head positioning systems [26]. In this case, the magnetic field is exerted in the perpendicular direction to the coil surface. For linear motion electromagnetic energy harvesters, where the permanent magnet moves inside the coil, deriving the equation for the induced voltage is difficult, mainly because of the spatially nonuniform distribution of the magnetic field [27].

For deriving the formula for the induced voltage, in that case, it is necessary to use the formula of force acting on a particle in the magnetic field. The rearranged equation takes the form of the commonly known Lorentz law, as follows:

(1) F=qE+q [v × B]

where ***E***—electric field intensity vector, ***B***—vector of magnetic flux density—magnetic induction, *q*—particle of electric charge, *v*—velocity of particle.

The induced voltage can be derived from the balance of the forces acting on the particles moving in the magnetic field at a constant speed. To simplify the calculation of the electric field and the integration process along the winding circuit, the transformation of the electric field matrix from a cartesian to a cylindrical coordinate system is necessary. Assuming that the motion of the permanent magnet exists only on *z* axis of the cylindrical system, and the coil wires are wound around that axis the voltage induced in the single wire of the coil has the following form:

(2) $$e_{\Theta }=-{2\pi \;rB}_{r}v_{z},$$

where *r*—radius of single wire of the coil, *B*_r_—magnetic flux density vector component in the *r* axis of the cylindrical coordinate system, *v*_z_—speed vector in the *z* axis of the Cartesian coordinate system.

**Figure 1:**
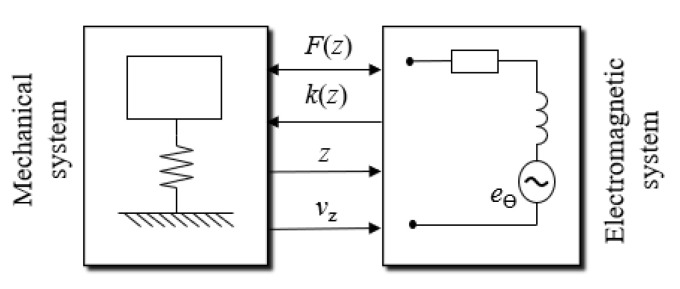
Model of an electromagnetic energy harvester.

Hence the electromagnetic energy harvester can be modeled as a two-part system: electromagnetic and mechanical (Figure 1). In this case, the velocity of the relative movement (displacement and the speed of the displacement) of the permanent magnet in a coil, regarded as a part of the mechanical system, establishes an input to the electromagnetic system and allows it to produce voltage. The electromagnetic system has an influence on the mechanical system—the magnetic field of two fixed magnets that limit the movement of the moveable magnet, which has an influence on the magnetic spring coefficient and the damping force that depends on the flux density, the number of the coil turns, the coil diameter and the magnetic field excited by the current flow in the coil when energy is harvested [28].

This paper analyzes the mechanical system consisting of a moveable permanent magnet, the tube with an adjustable distance in which the moveable magnet can move, and the part of the electromagnetic system consisting of magnetic springs without a coil (in which the voltage is induced). The magnetic spring allows the setting of the resonant frequency at desired values. The repulsive force between the magnets is a nonlinear restoring force due to the change of the field intensity depending on the distance of the magnet. The repulsive force is a derivative of potential energy, which depends on the magnet’s geometry and parameters and on the distance between them, therefore can be considered a nonlinear spring force [19]. This force of the magnetic spring was modeled using finite element methods (FEM) in the Comsol Multiphysics program.

**Figure 2:**
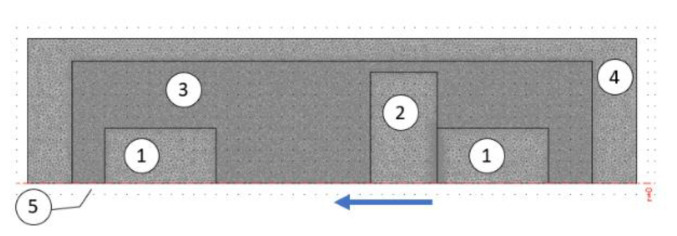
Axial symmetry of energy harvester geometry. Fixed permanent magnet of nonlinear magnetic springs—1, movable permanent magnet—2, free space (air gap)—3 and air—4, axial symmetry axis—5.

The energy harvester system has cylindrical axisymmetric geometry and therefore can be analyzed as a 2D axisymmetric problem. The real object—the magnetic spring was modeled while keeping its geometry. Therefore, in the model, there are two fixed cylindrical magnets with an initial diameter of 5 mm and height of 5 mm, which limits the range of the moveable magnet motion. Between them, a moveable cylindrical magnet is placed, which is 3 mm high and has a 10 mm diameter. The moveable magnet has a higher diameter in order to increase flux density and in consequence harvested energy and also to prevent rotating of the magnet while allowing adjustment of the air gap (fixed magnets are in the screw shaped tube, so the internal diameter of the tube is higher than 5 mm). The moveable magnet is able to move between the two fixed magnets in an 11 mm high air gap. The implemented axial geometry of the model of the energy harvester in Comsol program after meshing is shown in Figure 2. The initial position of the moveable magnet is near the right (bottom) magnet, and the gap between them is 10 μm wide. In the area of the two fixed magnets after meshing, there are about 4500 elements, in the area of the moveable magnet there are about 3000 elements, in the area of the air gap there are about 40,000 elements. Around the air area denoted by 3 Neumann Boundary Condition are applied, the line magnetic flux is normal to and passes through the boundary. The area around the airgap, where finally the coil or coils will be wound, consists of about 6000 elements. Around the air area denoted by 4 Dirichlet Boundary Condition are applied. The remanent magnetic flux density *B*_r_ is equal to 1.24 T.

**Figure 3:**
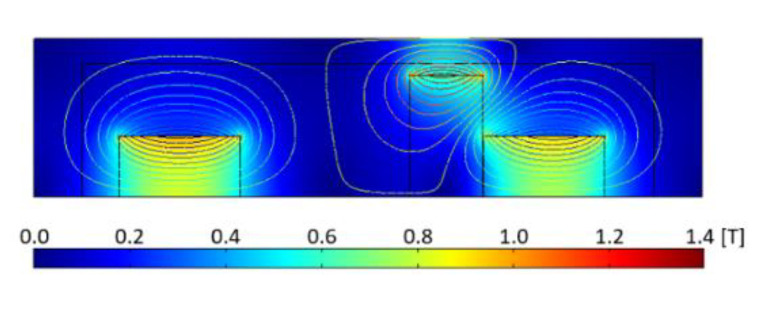
Flux density of the harvester when moveable magnet is near fixed magnet.

Simulations were made in a loop in the Comsol Script with 700 steps. Each step of the loop, consisting of calculations of the total energy of the area of the moveable magnet, was simulated after moving the moveable magnet about 10 μm from its previous position. The examples of the results representing the flux density are shown in Figure 3. The lines of the magnetic flux show that the magnets repel each other and reduce the flux density of each other.

The repulsive force acting on the moveable magnet from the fixed magnets was calculated from the differentiation of energy as follow:

(3) $$F_{z}(i)=\frac{W_{e}{(i)\;-\;W}_{e}\left(i+1\right)}{\delta \;z}$$

where *F*_z_(*i*)—repulsive force calculated in *i* step, *W*_e_(*i*)—total energy of the moveable magnet area calculated in *i* step, *W*_e_(*i* + 1)—total energy of the moveable magnet area calculated in *i* + 1 step, δ*z*—displacement of the moveable magnet (from *i* to *i* + 1 step) (10 μm).

For the analysis, there were chosen two different cases of shapes of the fixed magnets belonging to two sets *c*_1_(0,*α*) and *c*_2_(*β*, 4). Set *c*_1_(0,α) described by a function of “0” and “α” denotes: “0”—there is no hole in the internal part of the permanent magnet, “α”—only the external diameter will be changed in a discrete way. Set *c*_2_(*β*,8) described by a function of “*β*” and “8” denotes: “*β*”—there is a change in the hole diameter in the internal part of the permanent magnets, “8”—there is a constant external diameter of the permanent magnet equal to 8 mm.

Approximation of the repulsive force over 699 calculation nodes was carried out using high order polynomials. Choosing the approximation polynomial degree, a balance between the quality of approximation and the complexity of the final polynomial has been kept. The extremely good conformity between the simulation results and the approximation is obtained using 9th degree polynomials. The coefficients of the approximation polynomial for different cases of shapes of the fixed magnets are shown in Table 1 and Table 2. The general formula for the approximation polynomial is as follows:

(4) $$F_{e}{(z)=a}_{9}z^{9}{+a}_{8}z^{8}+\ldots +\;a_{2}z^{2}{+a}_{1}{z+a}_{0}$$

where *a_i_* ∈ {0, 1, 2,…, 8, 9}—coefficients of the approximation polynomial of 9th degree, *z*—position of the moveable magnet.

**Figure 4:**
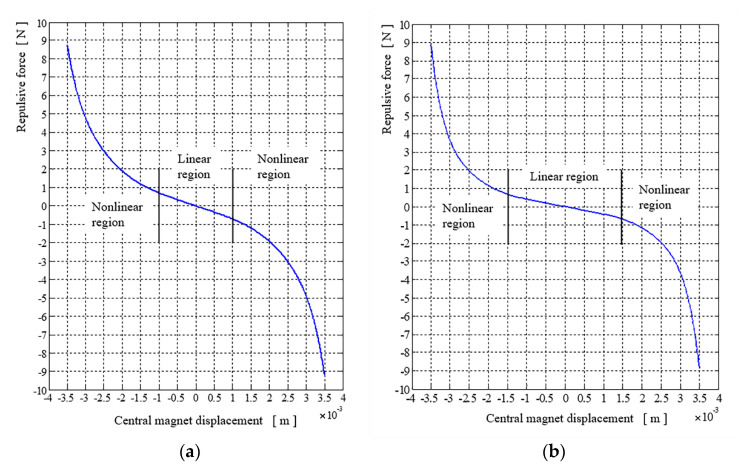
Repulsive force for magnetic spring *c*_1_(0,8) on the left (**a**) and on the right (**b**) for magnetic spring *c*_2_(4,8).

The exemplary results of the repulsive force versus the displacement of the movable magnet for the configuration case *c*_1_(0,8) and case *c*_2_(4,8) are presented in Figure 4.

In all these figures there are denoted regions where the repulsive force may be regarded as linear (or almost linear) and nonlinear (or strongly nonlinear). For energy harvesting purposes it is important to assure the free motion of the movable magnets in a vast range and possible high value of velocity, and forces exerted by the supporting spring (magnetic spring) should be small enough to build the desired resonant frequency close or equal to the external vibrations frequencies. To achieve that, it is necessary to obey four rules: a small repulsive force in the motion range (allowing for energy harvesting), enough linear range of repulsive force acting on the movable magnet (possibility of building the desired resonant frequency), a relatively small slope of the repulsive force in the motion region, a high repulsive force (in the nonlinear region) at the end of the designed range of motion (protection against crashing and potential energy storing for the retracting motion). The repulsive force generated in case *c*_1_(0,8), see Figure 4a, has a linear region spanned between ±1 mm, the slope of this region, calculated after the linear approximation of the data between this region, is equal *s*_1_ ≅ −675 N/m. The repulsive force generated in case *c*_2_(4,8), see Figure 4b, has a linear region spanned a little wider than in the previous case and it is spread between ±1.1 mm, but the slope of this region, calculated as previously, is equal *s*_2_ ≅ −374 N/m.

## 3. Stiffness of the Magnetic Spring and Laboratory Stand for Stiffness Testing

**Figure 5:**
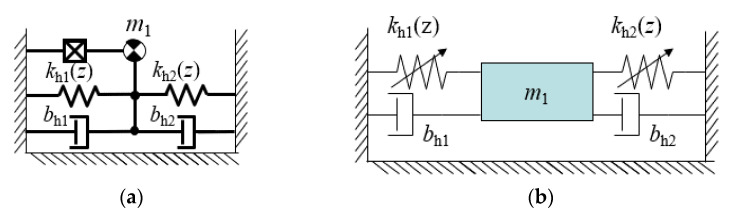
Magnetic spring as 1-DoF kinematic chain regarded as (**a**) robotic system (**b**) commonly known mechanical system.

After the FEM calculation and polynomial approximation of the repulsive force acting on the moveable magnet, the stiffness coefficient can be calculated by differentiation of the repulsive force with respect to the displacement of the moveable magnet. Knowing this coefficient, it is possible to represent the magnetic spring as a system consisting of a 1-DoF (Degree of Freedom) kinematic chain with a prismatic joint [26,29,30,31,32,33,34], which means springs supporting a mass. The mass can only move in a linear forward and backward direction. That assumption will simplify the calculation in the next steps. The mechanical part of the energy harvester system, which is a mass supported by magnetic springs, can be expressed in terms of a simple robotic chain with a prismatic joint, as is depicted in Figure 5a. Also, methods taken from robot dynamics can be applied to the mathematical model formulation [26,29,30,31,32,33,34], but it is also obvious that such a system may be represented by a well-known (from mechanical system modelling) diagram shown in Figure 5b. The formulation of a mathematical model of such an energy harvesting system in terms of a robotic system can be very useful when the energy harvester will be considered for operation with more a sophisticated system, like car tire energy harvesting systems [4,34], active shock absorbers powered by an energy harvester [4], power buoys [29,30], kinetic energy from human movement harvesting systems [5,8,16], etc.

**Figure 6:**
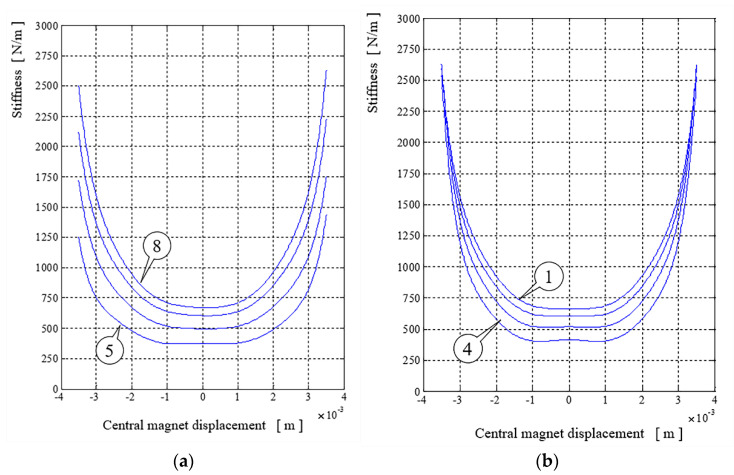
Stiffness of magnetic springs in geometric case *c*_1_(0,*α*)—(**a**) and *c*_2_(*β*,8)—(**b**), where numbers 1 and 4 indicates number of *β* and numbers 5 and 8 indicates number of *α*.

In Figure 6a we can see the set of curves representing the stiffness of the magnetic spring with the geometric configuration belonging to set *c*_1_(0,*α*). The stiffness curves resemble parabolas and differ in the whole range of the moveable magnet movement (±4 mm).

The set of curves representing the stiffness of the magnetic spring with the geometric configuration belonging to set *c*_2_(*β*,8), see Figure 6b, are crossing each other near the distances ±3.5 mm. In the expected motion range of the movable magnet (±1 mm), we can see that the curves representing the stiffness of the magnetic spring with the geometric configuration belonging to set *c*_1_(0,*α*) are nearly flat, and the smaller the diameter, the more flattened the curve. The curves representing the stiffness of the magnetic spring with the geometric configuration belonging to set *c*_2_(*β*,8) near that range are also flattened. However, for a larger hole, when the moveable magnet is in the central position, the stiffness is growing. The larger the hole, the higher the growth.

The stiffness coefficient *k* of the whole magnetic spring can be calculated from the force acting on the moveable magnet from Equation (4) divided by *z*. Equation (5) presents the stiffness coefficient dependence on the displacement of the moveable magnet:

(5) $$k_{h}{(z)=a}_{9}z^{8}{+a}_{8}{z\;}^{7}{+\ldots +a}_{2}{z+a}_{1}+\frac{a_{0}}{z}$$

where *a_i_* ∈{0, 1, 2,…, 8, 9}—coefficients of the approximation polynomial of 9th degree, *z*—position of the moveable magnet.

Two fixed magnets are identical, hence the stiffness coefficient of one spring between one of the fixed magnets and the moveable magnet is equal to half of the stiffness coefficient for the whole magnetic spring.

**Figure 7:**
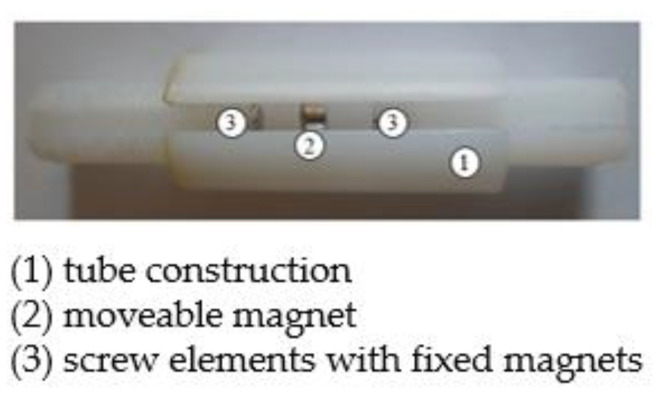
Prototype of energy harvester magnetic spring.

The last term in Equation (5) is small in comparison to others terms (see Table 1 and Table 2) and can be omitted. In a real manufactured prototype of the mechanical system of the energy harvester, the magnetic spring consists of two fixed magnets and one movable magnet between them. In the presented case two fixed magnets are 5 mm high and have a 5 mm diameter, and the movable magnet has a 10 mm diameter and is 3 mm high. The magnets are 4 mm away from each other. Figure 7 presents a manufactured prototype of the magnetic spring. The tube construction (denoted by 1) allows for free movement of the moveable magnet (denoted by 2). A gap in front of the tube allows to reduce the pressure inside and allows for observation and measurement of the moveable magnet displacement. The screw elements with fixed magnets (denoted by 3) allow for a change of the distance between the magnets or a change of these magnets or a moveable magnet, for example for a higher moveable magnet or magnets with other parameters characteristics.

**Figure 8:**
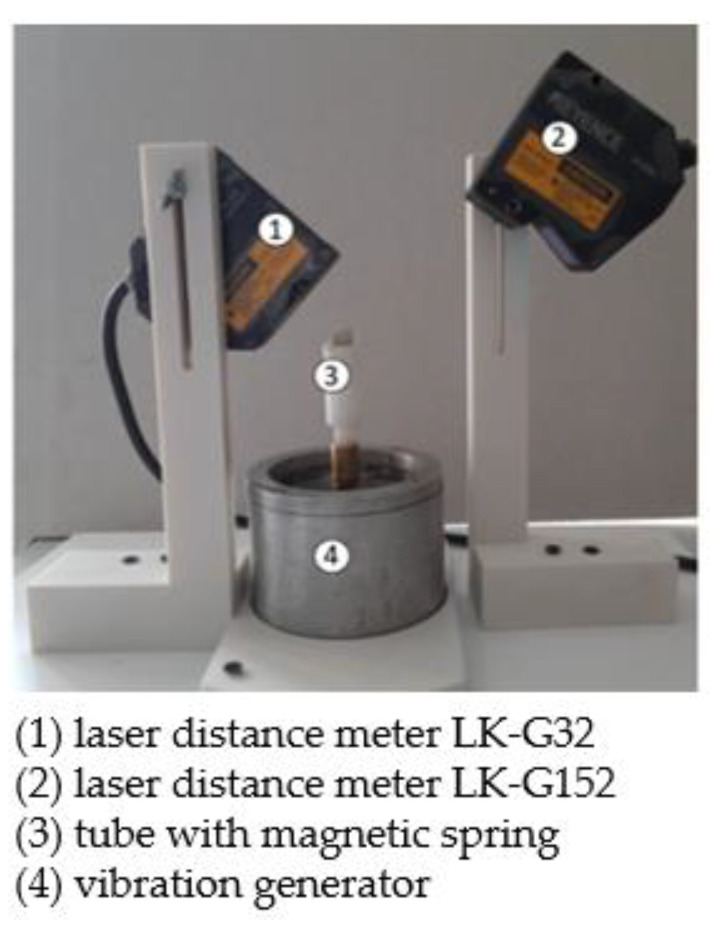
Measuring station for shaker and movable magnet motion under different current amplitude and frequency supply.

A measuring station was designed for the measurement of the position change of the movable magnet after a vibration exerted by the shaker (vibration generator), which is presented in Figure 8. For the permanent magnet motion measurement, two Keyence LK-G3000 series laser distance meters were used, which allow us to measure the displacement with very high accuracy. The first Keyence laser distance meter LK-G32 (denoted by 1) with repeatability equal to 0.05 μm was used for measurement of the vibration amplitude of the tube and the second laser distance meter LK-G152 (denoted by 2) with repeatability equal to 0.5 μm was used for measurement of the vibration amplitude of the moveable magnet. The sampling cycle for both laser meters was set at 200 μs. The vibration generator (denoted by 4) moves the tube with the magnetic spring (denoted by 3). The tube is fastened to the shaker mounting place in a very stiff manner and finally, the tube motion can be regarded as the shaker motion.

To determine the resonance frequency *f* (for an ideal linear system) it is necessary to calculate it from the stiffness coefficient and proof mass, as follows:

(6) $$f=\frac{1}{2\pi }\sqrt{\frac{k}{m}}\;$$

where *k*—stiffness coefficient of the spring, *m*—proof mass (in this case mass of the moveable magnet).

Based on Equation (6) it is possible to calculate the expected resonance frequency for the magnetic spring. Assuming that the moveable magnet is moving in a small range from its equilibrium position (±1 mm), it is possible to state that the stiffness coefficient is constant and it is equal to 375.4 N/m for this configuration of the magnetic spring. The movable magnet’s weight is 1.77 × 10^−3^ kg and after the initial assumption that the system can be considered linear, it is possible to estimate the resonance frequency as 73.36 Hz. The model should also take into consideration the parameters of the vibration generator to determine the exact resonance frequency. Equation (6) is simplified to the case where only the magnetic spring is considered. The amplitude of the generated sinusoidal signal was amplified by the amplifier IRS2092. Due to the nonlinear characteristic of the amplifier and the resultant distorted output voltage waveform, the input current amplitude was limited and kept constant. That also simplified modelling and simulation, because the force acting in the vibration generator can be expressed as a current multiplied by the so-called force constant *k*_t_.

**Figure 9:**
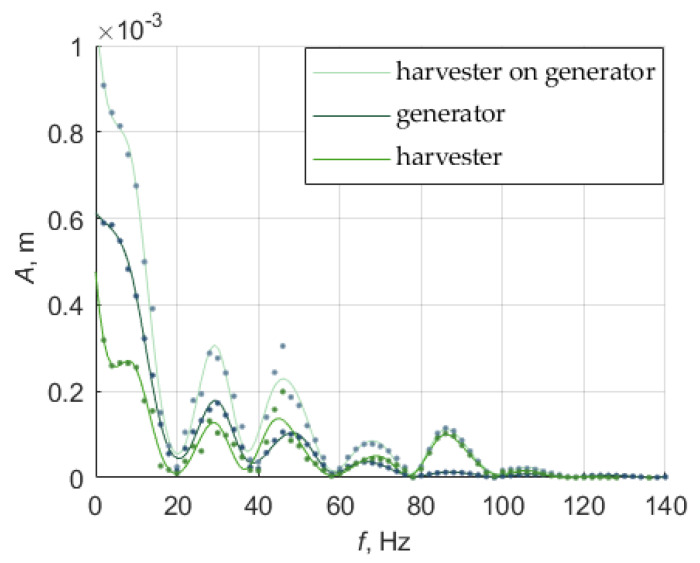
Amplitude-frequency graph for input current 1.2 A (where dots are real data and lines are approximation).

Results of the vibration amplitude for constant current 1.2A are presented in Figure 9. There are visible distinct increases of the amplitude of the harvester (it means only moveable part—magnet) and a vibration generator (with housing of the harvester) movement near 2 Hz, 30 Hz, 46 Hz, and 68 Hz and for the harvester itself near 86 Hz and 110 Hz. The increase of the amplitude of 30 Hz, 46 Hz, and 68 Hz could indicate the resonance frequency of the generator, which is constructed of two membrane springs and a rod between them. The first rise (2 Hz) can be caused also by the force from the supply current. Knowing that resonance frequency it is possible to calculate an approximation of the stiffness coefficient for membranes (planar springs) (without taking into account damping force). The exact stiffness coefficient was adjusted by the optimization process. The increase of the amplitude for the harvester near 86 Hz could indicate the approximated resonance frequency. In other rises of the amplitude harvester is following the movement of the generator. The results of the measurement and calculation of the resonance frequency of the harvester differ slightly. It may be caused by the approximation of the force acting on the moveable magnet.

## 4. Mathematical Model of Vibration Generator and Energy Harvester System Formulation

**Figure 10:**
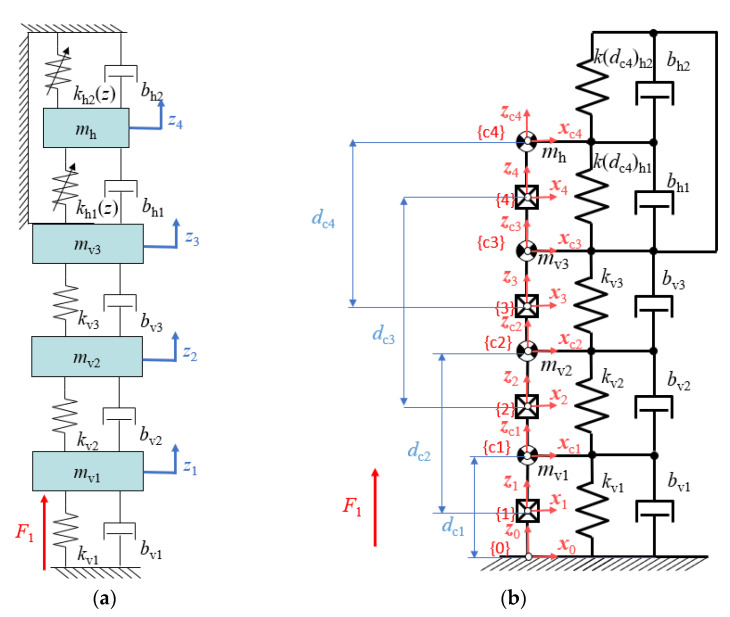
Kinematic chain of vibration generator and energy harvester expressed in: (**a**)—two springs mass mechanical system, (**b**)—robotic kinematic chain.

The system consisting of two fixed magnets and one moveable magnet can be represented as mass *m*_h_ between two springs with the stiffness coefficients *k*_h1_ and *k*_h2_ and dampers *b*_h1_ and *b*_h2_. The vibration generator consists of two planar springs of different shapes. Based on Figure 9 there is at least three different resonance frequency in these vibration generators, so it was assumed that the planar spring has 3-DoF, so there are three linear movements with three stiffness coefficients *k*_v1_, *k*_v2_, *k*_v3,_ and three masses *m*_v1,_ *m*_v2,_ *m*_v3_. It was assumed that with each of that movement there is damping force related, so the damping coefficients were *b*_v1_, *b*_v2_, *b*_v3_. That system can also be represented as a case of the robotic kinematic chain. These two representations are shown in Figure 10a,b respectively.

To determine the forces acting on the moveable magnet and the moveable part of the vibration generator and to describe the movements of these elements it is possible to use Lagrange’s equation (Equation (7)), which is one of the most common ways to determine the behavior of the system, as follows:

(7) $$\frac{d}{\mathrm{dt}}\frac{\partial E_{k}}{\partial \;\dot{q}}\;-\;\frac{\partial E_{k}}{\partial q\;}\;-\;\frac{\partial E_{p}}{\partial q}=F\;-\;\frac{\partial D}{\partial \;\dot{q}}$$

where *E*_k_—kinetic energy, *E*_p_—potential energy, *F*—external force, *D*—dissipation energy function, *q*—generalized displacement.

A model of such a system is presented in Figure 10a in which *z*_1_, *z*_2_, *z*_3_ are the positions of the elements of the vibration generator (namely: the vibrating head and the case of the analyzed harvester; because a planar spring having 3 natural frequencies was used in the construction of the generator, 3 degrees of freedom are imposed) and *z*_4_ is the moveable magnet position. Force *F*_1_ is the force of the vibration generator, it was assumed that is acting on a second mass of the vibration generator, and is given by:

(8) $$F_{1}{=k}_{t}i_{t},$$

where *k*_t_—force coefficient: product of flux density *B*, the number of coil turns *N* and length of one coil turn *l*, which is the product of 2π and radius *r*; *i*_t_—current applied to the coil of the vibration generator. The external force acting on mass *m*_h_ is in that case equal to zero.

Another way to describe the movement of the moveable magnet and vibration generator is using Denavit-Hartenberg notation and the Newton-Euler equation. In Figure 10b the system consisting of the energy harvester and vibration generator is shown as a kinematic chain with four prismatic joints, springs, and dampers between the masses (centers of mass). The prismatic joints represent the linear movement of the vibration generator moveable part and of the moveable magnet in the energy harvester. The kinematic parameters for the system consisting of the energy harvester and vibration generators, such as the length of the links and joint angles are 0 (the length of the links does not affect the matrixes in that linear case), except for the variable distances between the mass centre and the previous *x* axis *d*_c1_, *d*_c2_, *d*_c3_ and *d*_c4_, which are denoted as *d*_1_, *d*_2_, *d*_3_ and *d*_4_, accordingly, later in the equations.

The homogenous transformation is obtained from rotation and translation about the *x* and *z* axes. The homogenous transformation for the whole kinematic chain is the product of homogenous transformations *A* for each joint. In this case, the homogenous transformations for each joint are diagonal unitary matrixes with one additional element *d*_1_, *d*_2_, *d*_3_ or *d*_4_ in the fourth column of the third row.

To obtain equations of the forces acting on the system, matrix form of the Euler-Lagrange equation can be used. For the kinematic chain from Figure 10b it is shown below:

(9) $$D[{\ddot{d}}_{i}{\;]+C[\;\dot{d}}_{i}]+[F_{\mathrm{pi}}]=[F_{i}{-F}_{\mathrm{bi}}],$$

where ***D***—inertia matrix, ***C***—Christoffel matrix, *d*_i_—displacement of the mass of the *i* joint, *F*_pi_—potential force acting on *i* joint, *F_i_*—external force acting on *i* joint (only one external force acting on vibration generator (Equation (8)), *F*_bi_—damping forces acting on *i* joint.

Matrix ***D*** is an inertia matrix and is obtained as a part of a kinetic energy which is represented by the formula:

(10) $$E_{k}=\frac{1}{2}{\;\dot{q}}^{T}\sum_{i=1}^{n}{(m}_{i}J_{vci}^{T}{(q)J}_{vci}{(q)+J}_{\omega ci}^{T}{(q)R}_{i}{(q)I}_{i}R_{i}^{T}{(q)J}_{\omega ci}\left(q)\right)\dot{q\;}=\frac{1}{2}{\;\dot{q}}^{T}D(q)\;\dot{q},$$

where ***R****_i_* -a rotation matrix that enforces the formulation of rotational speed coordinates depending on link *i* coordinates, *m*_i_—mass of *i* link, ***I****_i_*—inertia matric evaluated around a coordinate axis parallel to frame *i*, ***q***—vector of generalized displacement, ***J***_v*i*_—Jacobian of the linear speed of *i* joint, ***J***_ω*i*_—Jacobian of the rotational speed of *i* joint.

Since each joint in the kinematic chain is prismatic, the Jacobian of the linear speed depends on *z* axis of *i*−1 joint, and the Jacobian of the rotation speed is 0. The Jacobians for each mass centre are 6×4 matrixes and their elements are equal to 0, except the third row, which is for the first joint [1 0 0 0], for the second joint [1 1 0 0], for the third joint [1 1 1 0] and for the last joint [1 1 1 1]. The matrix of inertia ***D*** is formulated as the sum of products of the mass and Jacobians of the linear and rotational speed.

(11) $$\;D=\left[\begin{array}{cccc} m_{v1}{+m}_{v2}{+m}_{v3}{+m}_{h} & m_{v2}{+m}_{v3}{+m}_{h} & {+m}_{v3}{+m}_{h} & {+m}_{h}\\ m_{v2}{+m}_{v3}{+m}_{h} & m_{v2}{+m}_{v3}{+m}_{h} & {+m}_{v3}{+m}_{h} & {+m}_{h}\\ m_{v3}{+m}_{h} & m_{v3}{+m}_{h} & m_{v3}{+m}_{h} & m_{h}\\ m_{h} & m_{h} & m_{h} & m_{h} \end{array}\right],$$

where *m*_v1_, *m*_v2_, *m*_v3_—masses attached to the corresponding part of the vibration generator (so that the sum *m*_v1_ + *m*_v2_ + *m*_v3_ is equal to the collective mass of the vibrating head and the housing of the harvester), *m*_h_—mass of the moveable magnet in energy harvester.

The elements of Christoffel’s matrix ***C*** are the differentials of matrix ***D*** elements with respect to displacement variables, so the elements of matrix ***C*** are all equal to 0.

Force *F*_pi_ are forces from potential energy: gravity energy and energy of springs. The forces of the spring can be calculated as a matrix of the spring stiffness multiplied by the displacement as follows:

(12) $$\left[\begin{array}{c} F_{p1}\\ F_{p2}\\ F_{p3}\\ F_{p4} \end{array}]=[\begin{array}{cccc} k_{v1} & 0 & 0 & 0\\ 0 & k_{v2} & 0 & 0\\ 0 & 0 & k_{v3} & 0\\ 0 & 0 & 0 & k_{h} \end{array}][\begin{array}{c} d_{1}-d_{10}\\ d_{2}-d_{20}\\ d_{3}-d_{30}\\ d_{4}-d_{40} \end{array}]+[\begin{array}{c} m_{v1}g\\ m_{v2}g\\ m_{v3}g\\ m_{h}g \end{array}\right]$$

where *d*_10_, *d*_20_, *d*_30_ and *d*_40_—initial positions calculated from gravity force divided by stiffness coefficient, for the magnet *d*_40_ the stiffness coefficient of the starting position was assumed to be equal to the coefficient *a*_1_ from Equation (5) (gravity forces and initial position multiply by the stiffness coefficients cancel each other); *k*_h_—stiffness coefficient from Equation (5) for whole magnetic spring; *k*_v1_, *k*_v2_, *k*_v3_—stiffness coefficients attached to the corresponding part of the vibration generator.

Damping forces for each joint movement depend on air resistance and mechanical damping of springs:

(13) $$\left[\begin{array}{c} F_{b1}\\ F_{b2}\\ F_{b3}\\ F_{b4} \end{array}\right]=\left[\begin{array}{cccc} b_{v1} & 0 & 0 & 0\\ 0 & b_{v2} & 0 & 0\\ 0 & 0 & b_{v3} & 0\\ 0 & 0 & 0 & b_{h} \end{array}\right]\left[\begin{array}{c} {\dot{d}}_{1}\\ {\dot{d}}_{2}\\ {\dot{d}}_{3}\\ {\dot{d}}_{4} \end{array}\right].$$

where *b*_v1_, *b*_v2_, *b*_v3_—damping coefficients attached to the corresponding part of the vibration generator, *b*_h_—damping coefficient of the energy harvester,

Equation (9) can be written as the matrix equation:

(14) $$\left[\begin{array}{c} {\ddot{d}}_{1}\\ {\ddot{d}}_{2}\\ {\ddot{d}}_{3}\\ {\ddot{d}}_{4} \end{array}\right]=\left[\begin{array}{cccc} \frac{1}{m_{v1}} & -\frac{1}{m_{v1}} & 0 & 0\\ -\frac{1}{m_{v1}} & \frac{m_{v1}+m_{v2}}{m_{v1}m_{v2}} & -\frac{1}{m_{v2}} & 0\\ 0 & -\frac{1}{m_{v2}} & \frac{m_{v2}+m_{v3}}{m_{v2}m_{v3}} & -\frac{1}{m_{v3}}\\ 0 & 0 & -\frac{1}{m_{v3}} & \frac{m_{v3}m_{h}}{m_{v3}m_{h}} \end{array}\right](\left[\begin{array}{c} F_{1}\\ 0\\ 0\\ 0 \end{array}\right]-\left[\begin{array}{c} F_{b1}\\ F_{b2}\\ F_{b3}\\ F_{b4} \end{array}\right]-\left[\begin{array}{c} F_{p1}\\ F_{p2}\\ F_{p3}\\ F_{p4} \end{array}\right]).$$

The movement of the moveable magnet and the generator can be obtained from the above equation. The position of the generator is the sum of the positions of the first three joints, i.e., *d*_1_, *d*_2_ and *d*_3_. The position of the moveable magnet is equal *d*_4_, and the position of the moveable magnet on the generator is the sum of the position of each joint.

The presented methodology leads to the equations describing the harvester system that are equivalent to those obtained using other methods and formalisms, e.g., Hamiltonian. The differences are found in the algorithm of the analysis of the system. The presented approach can be regarded as a mixture of strict definitions of consecutive geometrical coordinates describing the system (found in robotics) with the formulation of the equations based partially on kinetic energy (found in “energy-based” methods, i.e., Lagrange and Hamilton formalisms). Our method, while not as general and flexible because of not allowing to describe the system using freely chosen generalized coordinates, offers instead an algorithmic way of analysing a given harvester structure, which might be easier to track and adjust to the changes in the system if such a necessity occurs (e.g., due to a certain number of similar cases to be analysed). The described approach has also one formal advantage over the “energy-based” methods—it does not require the definition of the potential energy in the system as a function of generalized coordinates. This allows us to put forces acting on the system components—often nonlinear functions of the configuration of the harvester—directly into the equations. If the force is measured, as is often the case, it cancels the formal requirement of potential energy calculation.

**Figure 11:**
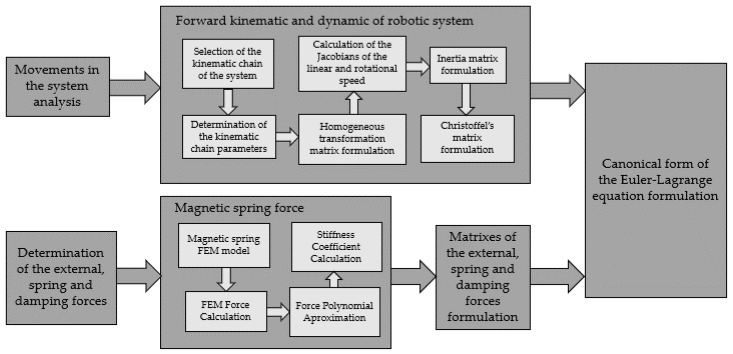
Graphic scheme of the steps of the methodology.

These methodologies can be presented in the flow diagram (Figure 11). The first step is analysis of all movements which occurs in the system and has influence on energy harvesting. Then it is possible to select the kinematic chain of the system, with joints that represent these movements. The kinematic chain is then described: joints and links are numerated, coordinate systems are attached to joints, and the kinematic chain parameters are determined. Based on kinematic chain parameters set out in the table homogenous transformation matrixes are formulated. From these matrixes, Jacobians of the linear and rotational speed for each joint are calculated. Jacobian of the speed for each joint allow Inertia matrix formulation, form which after parameters derivation by the variables (movements of the joints) Christoffel’s matrix is formulated. Determination of the forces: external, damping, or derived from the potential energy can be done in any moment of the previously described steps. In these cases also the calculation of the magnetic spring force should be added. After modeling the magnetic spring in the FEM program force acting on the moveable magnet can be calculated. Then force is approximated using polynomial approximation and the equation of the force is formulated. The stiffness coefficient is calculated as the derivative of the force by displacement of the magnet. After that, forces should be gathered in the matrixes.

## 5. Simulink Model of Magnetic Spring

**Figure 12:**
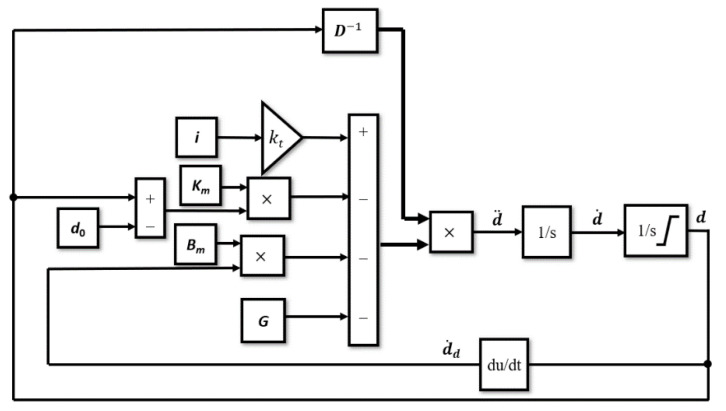
Model of the simulation of the movement of the moveable magnet in the magnetic spring on vibration generator.

A model of the magnetic spring on the vibration generator was made using Equations (8) and (12)–(14). Stiffness coefficients *k*_h1_ and *k*_h2_ are equal and are half of the stiffness coefficient from Equation (5), they depend on *d*_4_. Implementation of Equation (14) in Simulink/Matlab in the flowchart format is shown in Figure 12. In Figure 12 the block with input current *i_t_* contains a wave current from oscilloscope data. The current is amplified by coefficient *k*_t_ of the vibration generator in the gain block, coefficient *k*_t_ is calculated from Equation (15):

(15) $$k_{t}=BN2\pi \;r,$$

where flux density *B* = 0.7 T, coil turns number *N* = 50 and radius of coil *r* = 0.0185 m.

In the ***D***^−1^ block, the inversion of the inertia matrix ***D*** (Equation (11)) is calculated, as shown in Equation (14). The ***i*** block represents the vector of four elements: the second element is the current *i*_t_ from Equation (8), supplying the vibration generator, and the other elements are set to 0. In the ***K_m_*** block, the matrix with stiffness coefficients from Equation (12) is put. Lastly, the ***B_m_*** block contains a matrix with damping coefficients from Equation (13). Block ***K_m_*** is multiplied by vector ***d*** (contains each joint displacement) minus vector ***d***_0_ (contains each joint initial position). Block ***B_m_*** is multiplied by vector ${\dot{d}}_{d}$, which contains each joint velocity obtained by the time derivative of displacement ***d***. Block ***G*** contains a vector of gravitational force for each joint. Displacements of the joint are limited by the movement limitation of the magnetic spring and planar springs.

The current *i* is obtained from the scope that monitors the current supplying the vibration generator during measurements of the movement of the moveable magnet and the tube of the magnetic spring. It is assumed that the planar spring has three resonant frequencies, that occur respectively in a motion of 1/3 and 2/3 of its radius and in the center of the planar spring. Three masses of the planar spring are the results of that division. The mass *m_v_* of the moveable part of the vibration generator, i.e., planar springs, equals 0.32 kg, so the masses: *m*_v1_ equals 1/9 of *m*_v_, *m*_v2_ equals 3/9 of *m*_v_, *m*_v3_ equals 5/9 of *m*_v_. The moveable magnet mass *m*_h_ equals 1.77 × 10^−3^ kg. The stiffness and damping coefficients of the generator and damping coefficients of the magnetic spring are obtained by optimization process in the Matlab. Stiffness coefficient *k*_v1_ equals 10,064 N/m, *k*_v2_ equals 571 N/m and *k*_v3_ equals 5601 N/m. Damping coefficient *b*_v1_ equals 0.3211 Ns/m, *b*_v2_ equals 0.0243 Ns/m, *b*_v3_ equals 0.4115 Ns/m. The stiffness coefficient of the magnetic spring should be compared with the stiffness from Equation (5) for case *c*_1_(0,5) from Table 1. The damping coefficients of magnetic spring *b*_h1_ and *b*_h2_ are both equal to 0.0225 Ns/m. The displacements of the planar springs *d*_1_, *d*_2_, *d*_3_ are limited to ±10 mm and the displacement of the moveable magnet *d*_4_ is limited to ±4 mm.

## 6. Results

**Figure 13:**
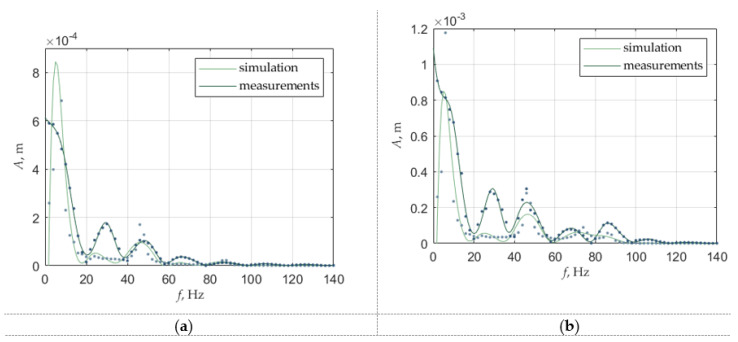
This amplitude-frequency graph for constant input current 1.2 A (**a**) for the tube (vibration generator) movement, (**b**) for the moveable magnet on the vibration generator movement (where dots are real data and lines are approximation).

Simulated and measured amplitude-frequency graphs for such a system are presented in Figure 13. The results of the measurements for the input current 1.2 A are compared to the results of the simulation (Figure 13). For the tube movement results are shown on the left side of the figures, denoted by (a), for the moveable magnet movement on the vibration generator results are shown on the right side of the figures, denoted by (b).

For the measurement, the moveable magnet movement is a result of differences between the movement of the harvester on the vibration generator and vibration generator movement. The movement of the moveable magnet is simulated as *d*_4_, the movement of the vibration generator was the sum of the displacement of the three first prismatic joints *d*_1_, *d*_2_, *d*_3_, and the movement of the moveable magnet on the vibration generator is a sum of all these movements—displacement of four prismatic joints.

The simulation results are highly dependent on the stiffness and damping coefficients of the springs. Dependencies between them may cause suppression of the resonance frequency or too high or too low amplitude compared to simulation results. The stiffness coefficient of the last prismatic joint related to the vibration generator (third) has an influence on the resonance frequency of the magnetic spring. The movement of the moveable magnet in a magnetic spring can be damped by that stiffness, but that stiffness has no influence on the value of the resonance frequency, which is about 74 Hz.

In the simulation results, it can be seen that the oscillations for certain frequencies are suppressed more than expected. To precisely model a vibration generator, more degrees of freedom or the planar spring could be considered and modeled as rotational joints, but the main movement is linear, so in these cases prismatic joints are sufficient. Additionally, in the model, the environment of the stand was not considered (the vibration generator is on the table and the vibration is also causing slight vibration of the table and the laser head). This inaccuracy however was slightly neglected by the optimization of the stiffness and damping coefficients. Displacement of the moveable magnet on the vibration generator for simulation follows the displacement of the tube up to about 35 Hz, and after that displacement of the moveable magnet is higher than the tube, like in measurements. In the lower frequency movement of the moveable magnet could be too damped in simulation. The resonance frequency for the moveable magnet differs from the measurement (74 Hz compared to 86 Hz). In the simulation, the moveable magnet has parameters for type N38, and for the actual measurements, these parameters could be slightly different. The force in the magnetic spring in measurement can differ also because of the misalignment between the moveable magnet and fixed magnets, due to rotation of the moveable magnets. Therefore, the stiffness coefficient can differ, so to improve the model the rotation and misalignment should be taken into consideration.

## 7. Conclusions

The energy harvester was modeled using the finite elements method (FEM) program Comsol Multiphysics. Field calculations were applied to calculate forces acting on the moveable magnet and therefore to determine the stiffness coefficient of the magnetic spring. The stiffness coefficient of the magnetic spring can be altered by changing the diameter of the fixed (external) magnets and therefore resonance frequency of the system can be altered. The larger the diameter, the higher the stiffness coefficient. In the range of the expected magnet movement (±1 mm), the curve is flattened. The holes in the fixed magnets are lowering the stiffness of the magnetic springs and are causing the magnet force-displacement characteristic to be convex on the flattened part of the stiffness curve. The stiffness, when the moveable magnet is in near proximity to the fixed magnets, has the same value for each hole diameter.

In the measurement results four resonant frequencies were identified, the resonant frequency of the vibration generator (about 30 Hz, 46 Hz, and 68 Hz) and the resonant frequency of the energy harvester (about 86 Hz).

The simulation model had been derived using the Denavit-Hartenberg notation. The behavior of the energy harvester placed on the vibration generator in this case can be modeled by four masses connected by prismatic joints and with springs and dampers for each joint. In the simulation the highest displacement of the moveable magnet was for the resonance frequency value calculated earlier, which means about 74 Hz.

The accordance between the derived model and the measurement results can be considered satisfactory, although there are some notable differences between the two. Those are caused by the differences between simulated force and a real force in a magnet, which is believed to be mainly caused by the misalignment and rotation of the magnets and also by the non-uniform magnetization and differences in the magnetic properties. To improve the model, the misalignment of the magnet should be taken into consideration. The model can also be improved by considering that the magnet in the energy harvester is not only moving in one direction (vertical movement) but is also rotating along its magnetization axis and along the axis perpendicular to the magnetization axis. However, the vertical movement of the magnet is the most significant for that type of energy harvester. The movement of the vibration generator could be approximated by the rotational joints and more degrees of freedom to improve the vibration generator model, because the planar spring can have more resonance frequencies.

The behavior of the energy harvester on the vibration generator can be represented by the kinematic chain with four or more degrees of freedom and derived from the Denavit-Hartenberg notation. That method is used to determine the motion of the working end of the manipulators in robotics and it is shown in these articles that it could be used also to determine the movement of the element that is harvesting energy, like a magnet in a magnetic spring from the electromagnetic generator. In consequence, such a model can be used to determine resonance frequency and as the result to optimize energy harvester for specific applications like a power supply for sensors e.g., in tire pressure monitoring systems.

In future research, the model will be improved by adding a rotational joint to the energy harvester. Due to the differences between simulated and real force the misalignment of the magnets and differences in magnetic properties will be further investigated. In order to determine energy harvested by such energy harvester induced voltage as well as the demagnetization process will be investigated.

## Figures and Tables

**Table 1:** Coefficients of 9th degree approximating polynomials for magnet case *c*_1_(0,α).

Polynomial Case	F1	F2	F3	F4
α [mm]	5	6	7	8
coeff	-	-	-	-
*a*_9_ × 10^23^	−1.259	−1.087	−1.270	−1.405
*a*_8_ × 10^19^	1.238	−1.548	−4.353	−4.797
*a*_7_ × 10^18^	2.389	1.941	2.291	2.473
*a*_6_ × 10^14^	−2.923	2.771	7.624	8.327
*a*_5_ × 10^13^	−1.803	−1.501	−1.813	−2.013
*a*_4_ × 10^7^	179.689	−163.1	−449.430	−495.030
*a*_3_ × 10^6^	14.757	−9.145	−15.957	−20.965
*a*_2_ × 10^1^	−441.790	2190	5079.3	5423.7
*a*_1_ × 10^2^	−3.754	−4.945	−6.062	−6.717
*a*_0_ × 10^−6^	−247.489	−3115.7	−10,676	−11,824

**Table 2:** Coefficients of 9th degree approximating polynomials for magnet case *c*_2_(β,8).

Polynomial Case	F5	F6	F7	F8
β [mm]	1	2	3	4
coeff.	-	-	-	-
*a*_9_ × 10^23^	−1.808	−2.077	−2.307	−2.611
*a*_8_ × 10^17^	4.123	−2.186	3.165	7.331
*a*_7_ × 10^18^	3.316	3.721	4.036	4.549
*a*_6_ × 10^12^	−6.948	4.620	−5.247	−13.041
*a*_5_ × 10^13^	−2.608	−2.860	−3.065	−3.373
*a*_4_ × 10^7^	3.211	−3.189	2.409	6.808
*a*_3_ × 10^6^	−3.258	8.666	21.527	37.861
*a*_2_ × 10^1^	−3.403	7.524	−2.747	−10.132
*a*_1_ × 10^2^	−6.606	−6.038	−5.169	−4.141
*a*_0_ × 10^−6^	−3.833	−32.219	0.658	16.246

## Data Availability

The data presented in this study are available on request from the corresponding author.
